# Overexpression of prothymosin-α in glioma is associated with tumor aggressiveness and poor prognosis

**DOI:** 10.1042/BSR20212685

**Published:** 2022-04-19

**Authors:** Anurag Kumar, Vikas Kumar, Mohit Arora, Manish Kumar, Prajwal Ammalli, Bhaskar Thakur, Jitender Prasad, Sarita Kumari, Mehar Chand Sharma, Shashank Sharad Kale, Shyam S. Chauhan

**Affiliations:** 1 Department of Biochemistry, All India Institute of Medical Sciences, New Delhi, India; 2 Department of Biochemistry, All India Institute of Medical Sciences, Bilaspur, India; 3 Department of Biochemistry, National Institute of Mental Health and Neuro-Sciences, Bengaluru, India; 4 Division of Biostatistics, Kalinga Institute of Medical Sciences, KIIT University, Bhubaneswar, India; 5 Department of Biochemistry, All India Institute of Medical Sciences, Deoghar, India; 6 Lab Oncology Unit, Dr. B.R.A-IRCH, All India Institute of Medical Sciences, New Delhi, India; 7 Department of Pathology, All India Institute of Medical Sciences, New Delhi, India; 8 Department of Neurosurgery, All India Institute of Medical Sciences, New Delhi, India

**Keywords:** GBM, Prognosis, PTMA, Survival

## Abstract

Prothymosin-α (PTMA), a nuclear protein, is strikingly associated with unfavorable clinical outcomes in many cancers. However, no information about its clinical relevance in glioma was available. Therefore in the present study, we evaluated the prognostic utility of this protein in a cohort of 81 glioma patients. The PTMA expression was assessed by immunohistochemical analysis, quantitative PCR, and Western blotting. Furthermore, the association of PTMA with clinicopathological features and molecular alterations were assessed in the patient cohort and validated in multiomics datasets, The Cancer Genome Atlas (TCGA; *n*=667) and Chinese Glioma Genome Atlas (CGGA; *n*=1013). We observed an increase in PTMA expression with increasing histological grades of this malignancy. PTMA immunostaining also displayed a strong positive association with the MIB-1 index. Univariate analysis revealed a superior prognostic value of PTMA to predict overall survival (OS) as compared with the routinely used markers (p53, isocitrate dehydrogenase (IDH) 1 (IDH1), α-thalassemia/intellectual disability syndrome X-linked (ATRX), and Ki-67). Interestingly, in Cox regression analysis it emerged as an independent predictor of OS (hazard ratio (HR) = 13.71, 95% CI = 5.96–31.52, *P*<0.0001). Thus, our results demonstrate the potential prognostic utility of PTMA in glioma which may prove useful in the management of this deadly malignancy.

## Introduction

Gliomas represent the vast constellation of malignant CNS tumors and a significant cause of cancer-related deaths worldwide [1]. Depending on the cellular origin, gliomas are classified as astrocytic, oligodendroglial, or oligoastrocytic, whereas histopathologically, gliomas are classified by the World Health Organization (WHO) into four grades; grade I–IV [2]. Among various glial cell types, astrocytic gliomas are the most common contributing to approximately 75% of all gliomas [3–5]. The treatment involves surgical resection, chemotherapy, most commonly temozolomide (TMZ), followed by radiotherapy [6]. Despite the latest interventions, only limited benefits are observed as overall survival (OS) remains dismally poor, especially for grade IV gliomas, commonly referred to as glioblastoma (GBM) [7]. Gliomas can arise in the brain *de novo* or evolve from lower grade astrocytoma. Furthermore, heterogeneity is also observed at the molecular level. Rapid recurrence after surgical resection and resistance to the therapy are significant challenges for current glioma treatment [8,9].

Molecular alterations in gliomas have been widely implicated in clinical decision-making [10,11]. Numerous studies have deciphered the loss of tumor suppressors and amplifications among the proto-oncogenes, which ultimately overwhelms apoptotic signals and accelerates multiple downstream proliferative pathways [12–15]. Mutation in the isocitrate dehydrogenase gene (*IDH*) and co-deletion of chromosome 1p and 19q (1p/19q co-deletion) in low-grade gliomas (grade I and II) are associated with favorable prognosis [16,17].

Prothymosin-α (PTMA) is a small (110–112 amino acids; 12.5 kDa), widely distributed acidic protein. It is a highly conserved multifunctional protein having striking pro-tumorigenic traits attributed to inhibition of apoptotic events and marked augmentation of cell division and pro-proliferative downstream signaling cascades [18–22]. Expression of PTMA correlates positively with oncoproteins/proliferation markers like Ki67 and c-myc [23,24]. Association of PTMA overexpression with unfavorable clinical outcomes has been observed in various cancer types, such as breast, head and neck oral squamous cell cancer, hepatocellular carcinoma, lung cancer, gall bladder cancer, colorectal cancer etc [20,25–30]. However, the involvement of this protein in glioma is yet to be established.

In the present study, we utilized publicly available multiomics glioma datasets and in-house clinical tissues to analyze the expression pattern of PTMA in glioma. We found that the expression of PTMA, both at mRNA and protein levels, was strongly correlated with the advancement of malignant histologic phenotypes in gliomas. Remarkably, we observed enhanced PTMA levels were associated with decreased survival time of glioma patients. Our data suggested that enhanced PTMA expression is a promising prognostic feature in this malignancy.

## Materials and methods

### Patients and tissues

For the present study, three cohorts of patients were used to describe the clinical significance of PTMA expression in glioma. Two of these cohorts were sourced from publicly available multiomics datasets, The Cancer Genome Atlas- lower grade glioma and glioblastoma (TCGA-LGG-GBM, n=667) and Chinese Glioma Genome Atlas (CGGA, n=1013). The third cohort included in-house patient samples (n=81). For this institutional cohort, brain tissue samples were taken from histologically confirmed gliomas operated on between March 2015 and September 2016 from adult patients below 65 years of age. These samples were collected from the Department of Neurosurgery, AIIMS (New Delhi, India), after the approval of the institutional ethical committee (reference number: IESC/T-244/05.05.2015), which followed guidelines recommended by the "Declaration of Helsinki". Preoperatively, written informed consent was obtained from each patient or their legitimately eligible representative as a mandatory requirement from the institutional ethical committee. These tumors were histologically segregated into four subtypes based upon WHO grading of gliomas (grade I–IV). Therapeutic interventions in these patients aimed at the maximal resection of tumor tissue without significantly impairing neurological functions. Post-operatively cycles of radiation and TMZ chemotherapy were utilized to counter residual malignancy. The tissue sample from each patient was fixed in liquid buffered formalin for immunohistochemical analysis. Additionally, a portion of freshly resected tissue was snapped frozen in liquid nitrogen for RNA isolation and western blotting experiments, wherever possible. A total of 81 histologically confirmed glioma samples were collected, which were formalin-fixed and paraffin-embedded along. Patient characteristics have been given in Table 1.

**Table 1:** Clinicopathological and molecular characteristics of the studied cohorts

		Institutional cohort (total = 81)		TCGA (total = 667)		CGGA (total = 1013)	
Characteristic	Group	Number of patients	Frequency (%)	Number of patients	Frequency (%)	Number of patients	Frequency (%)
**Age**	**≤45 years**	44	54.32	293	43.93	615	60.71
	**>45 years**	37	45.68	316	47.37	397	39.20
	**Not available**			58	8.70	1	0.1
**Gender**	**Male**	63	77.78	355	53.22	597	58.93
	**Female**	18	22.22	254	38.08	416	41.07
	**Not available**			58	8.70	-	
**Grade**	**I**	5	6.17	-	-	-	-
	**II**	35	43.21	226	33.88	291	28.73
	**III**	19	23.46	244	36.58	334	32.97
	**IV**	22	27.16	149	22.34	388	38.30
	**Not available**	-	-	48	7.20		
**Histology**	**Astrocytoma (A)**			194	29.09	389	38.40
	**Oligoastrocytoma (OA)**			130	19.50	30	2.96
	**Oligodendroglioma (OD)**			191	28.64	206	20.36
	**GBM**			152	22.80	388	38.30
**IDH mutation**	**Absent**			232	34.78	432	42.65
	**Present**			428	64.17	529	52.22
	**Not available**			7	1.05	52	5.13
**1p/19q co-deletion**	**Absent**			492	73.76	727	71.77
	**Present**			169	25.34	211	20.83
	**Not available**			6	0.90	75	7.40
**GBM subtypes**	**Classical**			58	38.16	106	27.32
	**Mesenchymal**			49	32.23	89	22.94
	**Proneural**			43	28.29	93	23.97
	**Not available**			3	1.97	100	25.77
**Radiotherapy**	**Not given**					162	15.99
	**Given**					765	75.52
	**Not available**					86	8.49
**Chemotherapy**	**Not given**					273	26.95
	**Given**					633	62.49
	**Not available**					107	10.56
**P53 immunostaining**	**Negative**	56	69.14				
	**Positive**	25	30.86				
**IDH immunostaining**	**Negative**	50	61.73				
	**Positive**	31	38.27				
**ATRX immunostaining**	**Negative**	30	37.04				
	**Positive**	51	62.96				
**PTMA immunostaining**	**Negative**	50	61.73				
	**Positive**	31	38.27				
**MIB1 index**	**Low (<10)**	52	64.20				
	**High (>10)**	29	35.80				

### Datasets used and data collection

We extracted RNA-seq data of glioma tissues from TCGA-LGG-GBM and CGGA studies. Patient characteristics of both the datasets along with the distribution of available clinical and molecular features have been given in Table 1. Also, microarray gene expression data from REMBRANDT (REpository for Molecular BRAin Neoplasia DaTa) (accession number: GSE108476, https://www.ncbi.nlm.nih.gov/geo/) was utilized to perform pathway analysis. The gene expression datasets were accessed through the GlioVis web server [31–33].

### Immunohistochemical analysis

The formalin-fixed and paraffin-embedded specimens were cut into 4-µm-thick sections. These sections were deparaffinized with xylene and subsequently rehydrated with a set of alcohol gradients. This was followed by heat-induced epitope retrieval by putting slides in Tris-EDTA buffer (0.01 M, pH 9:0) using a microwave oven. Thereafter, these slides were cooled to room temperature and incubated with 3% goat serum for 30 min to inhibit nonspecific binding, followed by incubation with the primary antibody in a humidified chamber at 4°C overnight. Slides were washed the following day with Tris-buffered saline (TBS) three times. To block endogenous peroxidase activity in the sections, they were treated with hydrogen peroxide (0.3% v/v in ethanol) for 20 min. Immunopositivity, as evident by positive chromogenicity with diaminobenzidine (DAB), was assessed using VECTASTAIN® Elite® ABC peroxidase kit following the manufacturer’s instructions. After this, counterstaining of the slides was done using Mayer’s Hematoxylin. The slides were then kept to dry overnight at room temperature, and then the next day, DPX was used as a mountant over which 22-mm coverslips were placed, avoiding air bubbles. These processed slides were then kept for letting DPX dry out, after which sections were analyzed using an inverted microscope [34,35] (Olympus BX51, U.S.A).

For the semiquantitative analysis of immunostaining pattern, immunoreactivity score (IRS) was calculated using staining intensity (I) and percentage of immunostained cells (P). For staining, I scores was from 0 to 3, where 0 was for no staining, 1 for weak or modest, 2 for moderate, and 3 for strong staining. Whereas for calculating the P score, we used the mean value of percentage immunopositivity in five randomly selected areas of tissue sections. Accordingly on the basis of percentage of staining, P score ranged from 0 to 4 and was categorized as: no staining or <10% of immunostained area, P = 0; 10–30%, P = 1; 30–50%, P = 2; 50–70%, P = 3; and >80%, P = 4. The final value of IRS was derived from adding I and P, the IRS (I + P) ranged from 0 to 7. The immunostaining analysis was assessed by an expert pathologist and his associates, who were blinded to the study variables. Analysis was performed based on the above criteria. To determine the association of PTMA protein levels with study variables, patients were assigned to PTMA-low (IRS 1–3) and PTMA-high (IRS 4–7).

### RNA extraction, cDNA preparation, and real-time PCR

Total cellular RNA was extracted using TRIzol reagent (Invitrogen, CA, U.S.A.) as per the manufacturer’s instructions from snapped frozen aliquots of brain tissues. One microgram of extracted RNA was reverse transcribed to cDNA using RevertAid reverse transcriptase kit (Thermo Fisher Scientific). qPCR was carried out using Maxima SYBR® Green (Bio-Rad Laboratories Inc., Hercules, CA, U.S.A.) in CFX96 Touch™ Real-Time PCR Detection System (Bio-Rad, Hercules, CA, U.S.A.) using specific primers for PTMA and β-actin gene as an internal reference gene for normalization. After the successful completion of the PCR cycle, a melting curve analysis was performed to ascertain the specificity of the PCR products. For quantitation of gene expression relative to a reference gene, the 2^−Δ*C*_t_^ method was employed. All the experiments were performed in triplicates. The following sets of primers were used for the amplification and relative quantitation of PTMA transcripts: PTMA forward primer 5′-ATGTCAGACGCAGCCGTAGACACCA-3′, reverse primer 5′-CTAGTCATCCTCGTCGGTCTTCTGC-3′; β-Actin forward primer 5′-CCTCGCCTTTGCCGATCC-3′ and reverse primer 5′-CGCGGCGATATCATCATC-3′. β-Actin gene was used as an internal control for normalization of the gene expression for PTMA.

### Western blotting

Tissue lysates were prepared from the glial tissues collected from the patients. All tissues were subjected to homogenization and sonication to obtain a clear lysate. Equal amounts (40 µg) of denatured protein lysates from tissues were resolved on to 15% SDS/PAGE. The proteins from the gel were transferred to a 0.2-μm PVDF membrane (10600021, GE Healthcare, IL, U.S.A.). This was followed by blocking with 3% bovine serum albumin or 5% non-fat dried milk for an hour. Subsequently, blots were incubated with goat polyclonal anti-PTMA (SC-18205, Santa Cruz Biotechnology, TX, U.S.A.) and mouse monoclonal anti-β-Actin (SC47778, Santa Cruz Biotechnology, TX, U.S.A.), which was followed by incubation with appropriate HRP-labeled secondary antibody. Lastly, ECL substrate (Pierce™ ECL Western Blotting Substrate, Thermo Fisher, MA, U.S.A.) was used to visualize the specific bands [36].

### Pathway analysis

Gene set enrichment analysis was performed for TCGA-GBM and REMBRANDT datasets, both of which were generated on the microarray gene expression platform. For this, a differential expression module of the GlioVis webserver was used. KEGG pathway and Gene Ontology were performed using gene expression cut-off as log_2_ fold change of 0.5, *P*-value and q-value cut-off were set at 0.05.

### DNA methylation analysis

Default parameters of the MEXPRESS web server (https://mexpress.be) were used to determine the Pearson correlation between the DNA methylation of PTMA promoter and its mRNA expression in TCGA-LGG and GBM datasets [37,38]. This web server uses gene expression, copy number variation, and DNA methylation data of TCGA dataset. DNA methylation data of the TCGA-LGG-GBM study were developed on the ‘Infinium HumanMethylation450 BeadChip’ platform. The predesignated methylation probes for PTMA were used for analysis.

### Survival analysis

Kaplan–Meier survival analysis for the in-house patient cohort was performed along with a log-rank test using the KM plotter web server (https://kmplot.com) with custom data. For survival analysis, patients were grouped according to high or low gene expression from the median; in contrast, for Cox proportional hazard model, gene expression was taken as a continuous variable to ensure the increased study power. The proportionality hazard assumption was assessed by using the Schoenfeld and scaled Schoenfeld residuals, and we found that there is no violation of the proportionality assumption. For in-house patients, all survival analyses were performed between two groups based on PTMA IRS. Information of OS, disease-specific survival (DSS), progression-free interval (PFI), and the disease-free interval (DFI) was available for TCGA-LGG and TCGA-GBM datasets, while for the CGGA dataset, only data for OS were available [39].

### Statistical analysis

Patients were followed-up for 3 years from the date of surgery for death. To determine the association of PTMA expression with clinicopathological features in the in-house cohort, we categorized the patients into two groups based on their PTMA IRSs obtained by immunohistochemistry. All patients whose IRS was up to 3 were designated as Low-PTMA IRS group, and remaining patients whose IRS was more than that (3–7) were segregated to High-PTMA IRS group. Pathological and clinical data were obtained from patient records of the Department of Pathology and the Department of Neurosurgery, respectively. Data analysis was performed using GraphPad Prism (version 6) and R-3.6.3. Fisher’s exact test was used to calculate the association between the categorical variables. For comparing gene expression of PTMA among different groups based on clinicopathological features, Mann–Whitney U-test was used. For all analysis, *P*-value <0.05 was considered as statistically significant (**P<0.001; **P<0.01; *P<0.05; ns, *P*>0.05).

## Results

### Expression pattern of PTMA in glioma

**Figure 1:**
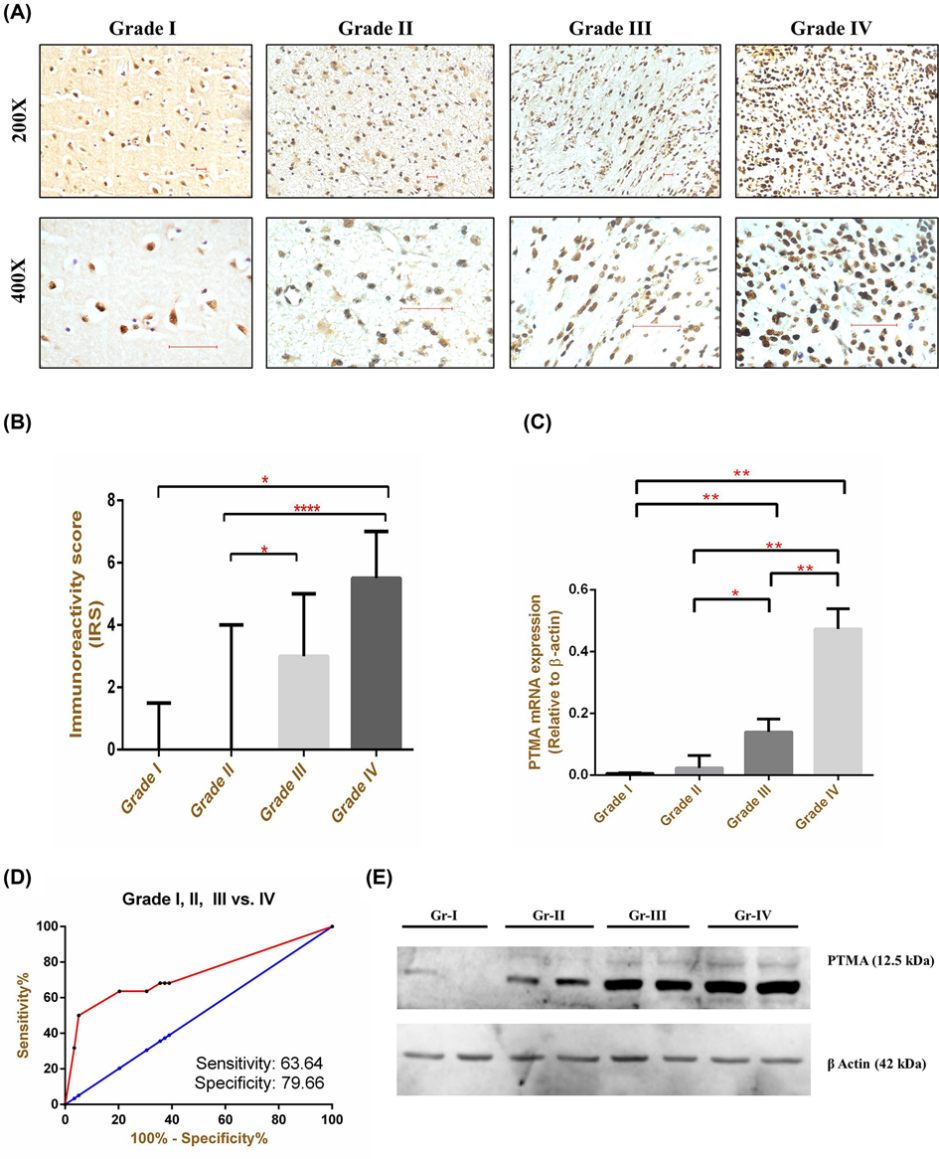
Expression of PTMA in glioma tissues (**A**) Immunostaining patterns in different glioma grades. (**B**) IRS among different grades of glioma. (**C**) mRNA expression of PTMA in representative glioma tissues (*n*=5, each grade, respectively). (**D**) Receiver operating characteristic curve for GBM diagnosis using PTMA IRS. (**E**) Total PTMA protein levels in representative glioma tissues from different grades. *P<0.05, **P<0.01, ****P<0.0001.

**Figure 2:**
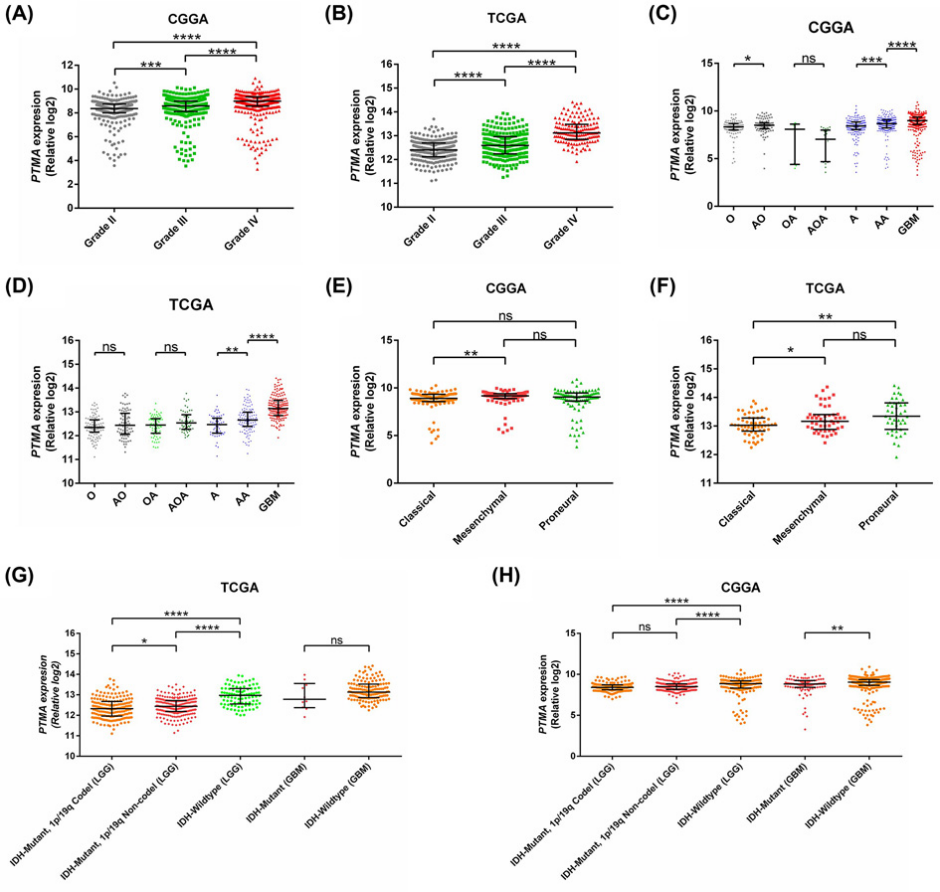
Expression of PTMA in CGGA dataset (left panel) and TCGA dataset (right panel) (**A**,**B**) Different grades of glioma; (**C**,**D**) histological subtypes; (**E**,**F**) molecular subtypes of GBM; (**G**,**H**) IDH mutation and 1p19q co-deletion. *P<0.05, **P<0.01, ***P<0.001, ****P<0.0001.

To determine the expression pattern of PTMA in glioma tissues, we performed immunohistochemistry for PTMA in different grades of glioma tissues from the in-house cohort, which revealed strong nuclear positivity of PTMA in advanced grades of glioma compared with lower grades (Figure 1A,B). Of the 81 histologically confirmed cases of glioma, the overall positivity rate for PTMA immunostaining was 47% (38/81); 40% (2/5) for grade I, 28.6% (10/35) for grade II, 57.9% (11/19) for grade III, and 68.2% (15/22) for grade IV. Further, overexpression of PTMA was also validated at the mRNA levels in representative tissues (Figure 1C). The receiver operating characteristic (ROC) curve for diagnosis of GBM from other grades using PTMA immunoreactivity revealed high sensitivity and specificity of 63.64 and 79.66, respectively (Figure 1D). The specificity of observed higher PTMA expression in the immunostaining data was also validated by Western blot in representative glioma tissues (Figure 1E). For a detailed exploration of aberrant PTMA expression in glioma, we utilized authoritative gene expression data of TCGA and CGGA studies. Comparison of PTMA gene expression among different glioma grades also revealed overexpression of PTMA in higher grade glioma (Figure 2A,B) in both TCGA and CGGA datasets. Further, the up-regulation was consistent when comparing astrocytoma (grade II) vs. anaplastic astrocytoma (grade III) (Figure 2C,D). Similarly, GBM tissues exhibited higher PTMA expression compared with anaplastic astrocytoma (grade III) (Figure 2C,D).

### Association of PTMA expression with clinicopathological and molecular features

The association of PTMA IRSs with clinicopathological features has been provided in Table 2. This revealed a strong association of higher PTMA level with MIB-1 index, a marker for cellular proliferation. For TCGA and CGGA datasets, we also compared its mRNA expression among recently described molecular subtypes of glioma [40], in which inconsistency existed between two datasets for PTMA expression pattern (Figure 2E,F). While the association between PTMA immunoreactivity and IDH mutation detected by immunostaining was not significant (Table 2), we observed in gene expression datasets, PTMA consistently exhibited higher expression in IDH-wildtype tumors compared with IDH-mutant tumors (Figure 2G,H). Among IDH mutant gliomas in the TCGA dataset, tumors that harbor 1p19q co-deletion exhibited reduced expression compared with tumors without it, while no such association was observed in the CGGA dataset (Figure 2G,H). No association of PTMA expression was observed with age or gender (Table 2, Supplementary Figure S1). PTMA mRNA expression was not associated with EGFR mutation, TP53 mutation, and PTEN mutation in GBM tissues from the TCGA dataset (Supplementary Figure S2).

**Table 2:** Association of PTMA immunostaining pattern with clinicopathological variables in the in-house cohort

Parameter		PTMA low	PTMA high	Fisher’s exact *P*-value
Gender	Male	37	26	0.412
	**Female**	13	5	
**Age**	**<45**	30	14	0.252
	**>45**	20	17	
**Grade**	**I**	5	0	0.018
	**II**	26	9	
	**III**	10	9	
	**IV**	9	13	
**p53 mutation**	**Absent**	38	18	0.137
	**Present**	12	13	
**IDH1 mutation**	**Absent**	28	22	0.241
	**Present**	22	9	
**ATRX mutation**	**Absent**	17	13	0.488
	**Present**	33	18	
**MIB-I index**	**Low (<10)**	39	13	0.002
	**High (>10)**	11	18	

Prognostic significance of PTMA in glioma. Abbreviation: ATRX, α-thalassemia/intellectual disability syndrome X-linked.

**Figure 3:**
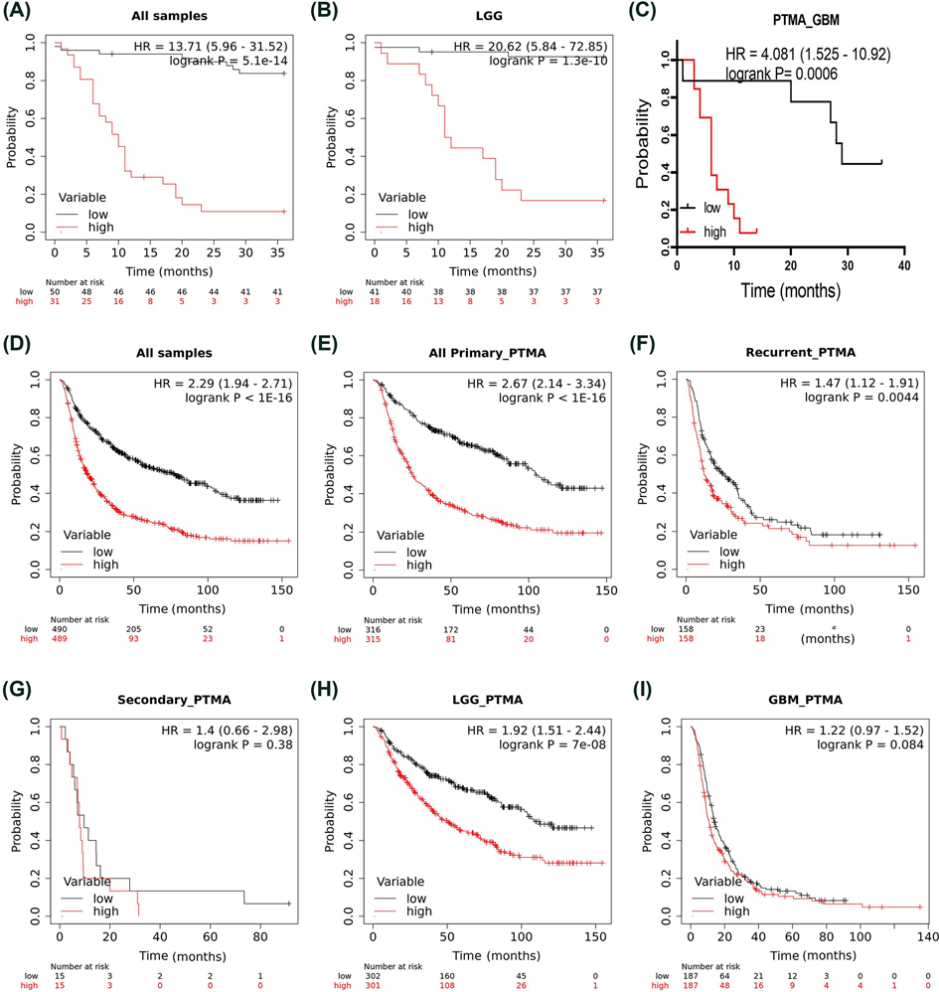
Association of PTMA expression with patient survival A-C and D-I represent the AIIMS cohort and CGGA dataset cohort, respectively. (**A**) All grades combined, (**B**) LGG, (**C**) GBM, (**D**) pan glioma analysis, (**E**) primary glioma tissues, (**F**) secondary glioma tissues, (**G**) recurrent glioma tissues, (**H**) LGG in CGGA, (**I**) GBM in CGGA. Abbreviation: GBM, glioblastoma multiforme. In all the panels, the low and high variable refers to low and high PTMA expression, respectively.

To determine associations of PTMA expression with patient survival in glioma, we performed Kaplan–Meier survival analysis for PTMA IRS in the patient data of our cohort with 36 months of follow-up for OS. Analysis of all samples revealed a higher PTMA level is associated with poor OS (hazard ratio [HR] = 13.71, 95% CI = 5.96–31.52, *P*<0.0001, Figure 3A). Further, similar results were obtained when the prognostic significance of PTMA was evaluated in LGG (HR = 20.62, 95% CI = 5.84–72.85, *P*<0.0001, Figure 3B), and GBM (HR = 4.08, 95% CI = 1.52–10.92, *P*<0.001, Figure 3C), separately.

**Figure 4:**
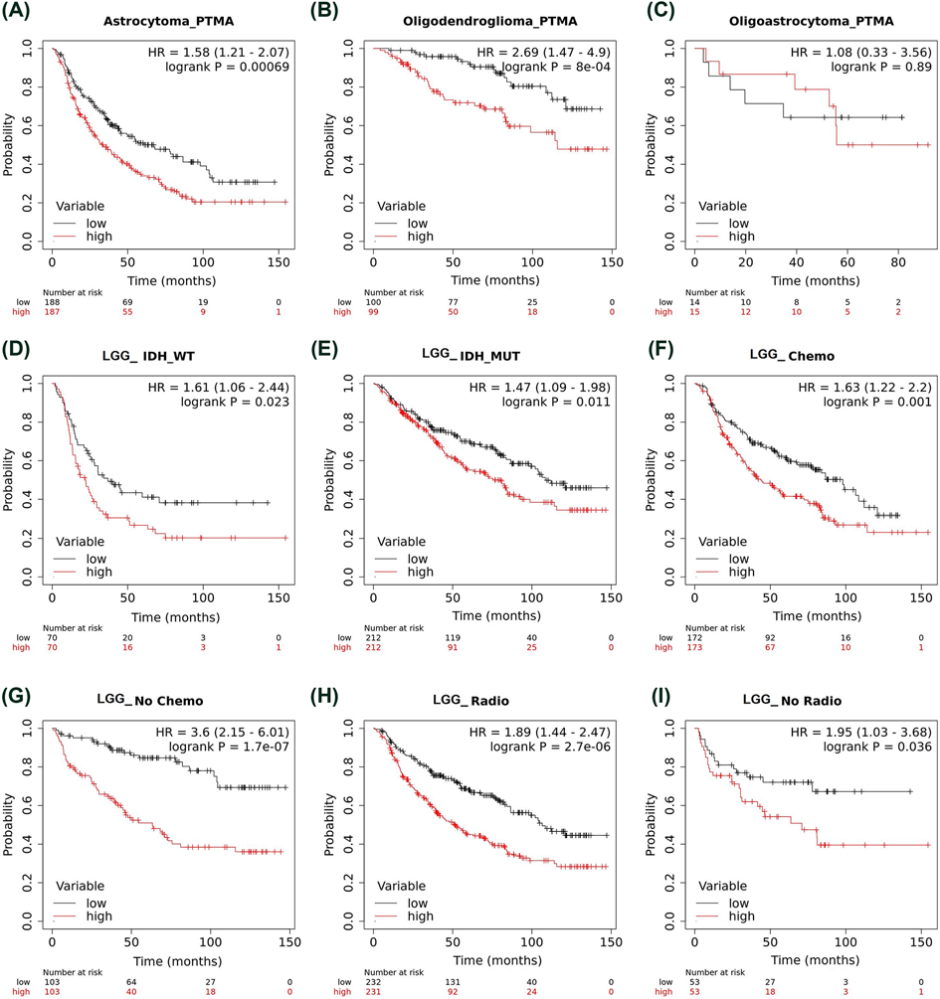
Association of PTMA expression and patient survival in lower-grade glioma (grade II + III) from CGGA dataset (**A**) Astrocytoma, (**B**) oligoastrocytoma, (**C**) oligodendroglioma, (**D**) IDH wildtype glioma, (**E**) IDH mutant glioma, (**F**) patients treated with chemotherapy, (**G**) patients treated without chemotherapy, (**H**) patients treated with radiotherapy, (**I**) patients treated without radiotherapy. Abbreviation: GBM, glioblastoma multiforme. In all the panels, the low and high variable refers to low and high PTMA expression, respectively.

Additionally, we assessed survival data of larger cohorts from CGGA and TCGA studies. For CGGA study data, Kaplan–Meier survival analysis in pan glioma analysis revealed that higher PTMA mRNA expression is associated with poor OS in CGGA dataset (HR = 2.29, 95% CI = 1.94–0.46, *P*<0.0001, Figure 3D). Similar association of higher expression of PTMA with poor prognosis was observed for primary glioma (HR = 2.67, 95% CI = 2.14–3.34, *P*<0.0001, Figure 3E), recurrent glioma (HR = 1.47, 95% CI = 1.12–1.91, *P*=0.0044, Figure 3F) but not with secondary glioma (HR = 1.4, 95% CI = 0.66–2.98, *P*=0.38, Figure 3G). Further, higher PTMA mRNA expression was associated with poor OS in LGG (HR = 1.92, 95% CI = 1.51–2.44, *P*<0.0001, Figure 3H) but not in GBM (HR = 1.22, 95% CI = 0.97–1.52, *P*=0.084, Figure 3I). We also performed subgroup-specific analysis of prognostic association of PTMA in CGGA dataset. In histology centered analysis, higher PTMA expression was associated with poor patient prognosis in astrocytoma (HR = 1.58, 95% CI = 1.21–2.07, *P*<0.001, Figure 4A) and oligodendroglioma (HR = 2.69, 95% CI = 1.47–4.9, *P*<0.0001, Figure 4B), but not in oligoastrocytoma (HR = 1.08, 95% CI = 0.33–3.56, *P*=0.89, Figure 4C). Further, among LGG, which includes grades II and III, similar association of higher PTMA expression was observed among IDH wildtype (HR = 1.61, 95% CI = 1.06–2.44, *P*=0.023, Figure 4D) and IDH mutant glioma (HR = 1.47, 95% CI = 1.09–1.98, *P*=0.011, Figure 4E), respectively. In context of therapy, the prognostic association of higher PTMA was observed with either presence or absence of chemotherapy (HR = 1.63, 95% CI = 1.22–2.2, *P*=0.001, Figure 4F; HR = 3.6, 95% CI = 2.15–6.01, *P*<0.0001, Figure 4G, respectively). Similarly, higher PTMA was also associated with poor OS in either with radiotherapy (HR = 1.89, 95% CI = 1.44–2.47, *P*<0.0001, Figure 4H) or without radiotherapy (HR = 1.95, 95% CI = 1.03–3.68, *P*=0.036, Figure 4I).

In the TCGA-LGG dataset also, the association of higher PTMA expression was observed with poor OS (Supplementary Figure S3A), DSS (Supplementary Figure S3B), PFI (Supplementary Figure S3C), but not with DFI (Supplementary Figure S3D). Stratifying patients based on IDH mutation suggested no association of PTMA expression with patient survival in either IDH wildtype (Supplementary Figure S3E–H) or IDH mutant glioma (Supplementary Figure S3I–L), suggesting that prognostic association of PTMA with patient survival is likely to be dependent upon the IDH mutation status. In astrocytoma tissues from the TCGA-LGG dataset, PTMA expression was associated with poor OS, DSS, and PFI, but not with DFI (Supplementary Figure S4A–D). Furthermore, in oligodendroglioma, higher PTMA was associated with poor OS, DFI, and PFI, but not with DSS (Supplementary Figure S4E–H). No association of PTMA expression was observed with the analyzed four survival parameters in oligoastrocytoma (Supplementary Figure S4I–L). In TCGA-GBM tissues, PTMA expression was not associated with OS, DSS, or PFI. Results of detailed prognostic parameters of PTMA expression in institutional and CGGA cohorts are given in Table 3. Intrestingly, multivariate analysis also suggested that higher PTMA expression is independently associated with poor patient prognosis in both institutional cohort (HR = 34.481, 95% CI = 10.231–116.206, *P*<0.001) and CGGA datasets (HR = 1.65, 95% CI = 1.37–1.99, *P*<0.001).

**Table 3:** Association of PTMA expression with OS in institutional and CGGA cohorts

		AIIMS cohort					CGGA dataset				
		Univariate		Multivariate				Univariate		Multivariate	
Characteristics		HR (95% CI)	*P*-value	HR (95% CI)	*P*-value	Variable	Category	HR	*P*-value	HR	*P*-value
**Age**		1.024 (0.999– 1.050)	0.057	1.004 (0.976–1.034)	0.761	**Age**		1.03 (1.02–1.04)	<0.001	1.01 (1.00–1.02)	<0.001
**Gender**	**Male**	Reference				**Gender**	**Male**	Reference			
	**Female**	0.669 (0.277–1.615)	0.371	1.492 (0.550–4.046)	0.431		**Female**	1.02 (0.87–1.21)	0.787	0.90 (0.75–1.08)	0.285
**WHO Grade**	**I**	Ref	to			**WHO Grade**	**Grade II**	Reference			
	**II**	3.67e+08	to	9.03e+07	to		**Grade III**	2.82 (2.18–3.64)	<0.001	2.67 (1.98–3.60)	<0.001
	**III**	9.94e+08 (3.83e+08 to 2.58e+09)	<0.001	3.36e+08 (1.14e+08 to 9.91e+08)	<0.001		**Grade IV**	7.92 (6.171–10.16)	< 0.001	4.55 (3.28–6.32)	< 0.001
	**IV**	2.14e+09 (9.19e+08 to 5.00e+09)	<0.001	1.34e+09 (3.46e+08 to 5.15e+09)	<0.001						
**P53 IHC**	**Absent**	Reference				**Tumor status**	**Primary**	Reference			
	**Present**	1.867 (0.941–3.699)	0.074	0.870 (0.390–1.941)	0.735		**Recurrent**	2.10 (1.77–2.45)	06	2.33 (1.92 –2.83)	<0.001
**IDH1 IHC**	**Absent**	Reference					**Secondary**	4.70 (3.20–6.89)	<0.001	2.80 (1.77 –4.42)	<0.001
	**Present**	0.662 (0.322–1.360)	0.261	1.550 (0.687–3.497)	0.291	**Histology**	**Astrocytoma/oligoastrocytoma (Ref)**	Reference			
**ATRX IHC**	**Absent**	Reference					**Oligodendroglioma**	0.28 (0.21–0.39)	<0.001	0.65 (0.39–1.07)	0.096
	**Present**	0.960 (0.481–1.919)	0.90	1.545 (0.617–3.869)	0.353		**GBM**	3.05 (2.55–3.63)	<0.001	4.55 (3.28–6.32)	<0.001
**MIB to 1 IHC score**		1.031 (1.018–1.044)	<0.001	0.986 (0.962–1.010)	0.259	**IDH/1p19q co-deletion**	**IDH wildtype, 1p/19q noncodel/codel (Ref)**	Reference			
**PTMA**	**Low**	Reference					**IDH mutant, 1p/19q noncodel**	0.48 (0.40–0.58)	<0.001	0.75 (0.59–0.94)	0.014
	**High**	15.683 (6.535–37.638)	<0.001	34.481 (10.231–116.206)	<0.001		**IDH mutant, 1p/19q codel**	0.14 (0.10–0.19)	<0.001	0.35 (0.21–0.59)	<0.001
						**Radiation status**	**Negative**	Reference			
							**Positive**	1.04 (0.82–1.31)	0.736	0.84 (0.65–1.08)	0.181
						**Chemotherapy status**	**Negative**	Reference			
							**Positive**	1.54 (1.27–1.87)	<0.001	0.67 (0.53–0.85)	0.001
						**PTMA expression**		1.49 (1.34–1.67)	<0.001	1.65 (1.37–1.99)	<0.001

Abbreviation: ATRX, α-thalassemia/intellectual disability syndrome X-linked.

### PTMA associated molecular pathways in glioma

**Figure 5:**
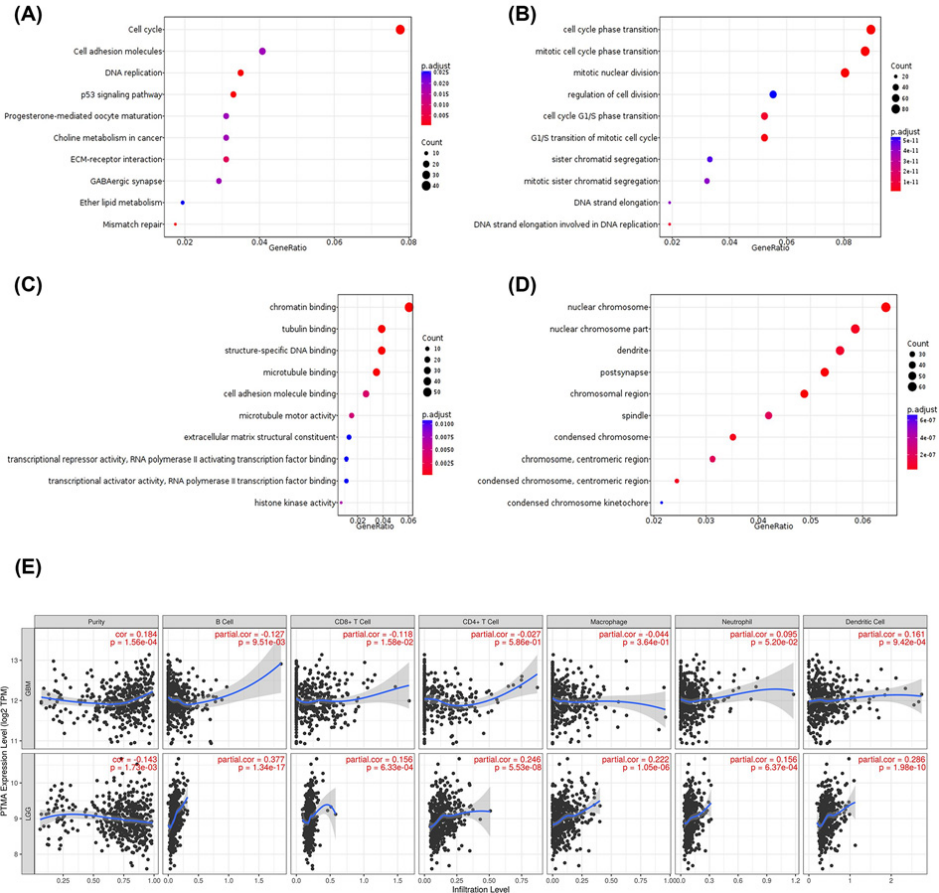
Pathway analysis of PTMA-correlated genes in glioma (**A**) KEGG pathway analysis, (**B**) Gene ontology: biological process, (**C**) Gene ontology: molecular functions, (**D**) Gene ontology: Cellular component, (**E**) TIMER analysis for the association between PTMA expression and immune cell composition in glioma.

To determine the molecular pathways associated with PTMA expression, we performed pathways analysis using Gene ontology and KEGG pathway analysis methods. KEGG pathway analysis revealed the association of PTMA expression with cell cycle, DNA replication, p53 signaling pathway, extracellular matrix to receptor interaction (Figure 5A). Gene ontology analysis also suggested the association of PTMA expression with cell cycle-related processes, chromatin binding, cell adhesion, and pathways occurring in the nucleus (Figure 5B–D). Furthermore, in light of its established roles in immunomodulation, we also determined the correlation of PTMA expression with infiltration of six different immune cells in the glioma microenvironment, separately for LGG and GBM in the TCGA datasets using TIMER webtool. This revealed that in LGG, higher PTMA expression is positively correlated with infiltration of all six immune cells (Figure 5E), while in GBM, it exhibited a positive correlation only with dendritic cells (*P*<0.001, Figure 5E).

## Discussion

PTMA is a nuclear oncoprotein transcription factor that has been known to harbor several pro-tumorigenic traits [41]. This is well evidenced by its elevated expression in robustly dividing cells while reduced in quiescent cells [42]. Functionally, PTMA is known to be highly pleiotropic. In the nucleus, PTMA affects the transcription of several genes like estrogen receptors by binding to histone H1 [43,44]. In the cytoplasmic compartment, PTMA mediates anti-apoptotic functions by blocking the caspase-9 activation which in turn inhibits apoptosome formation [22]. Interestingly, PTMA is also secreted extracellularly, where it acts as a damage-associated molecular pattern (DAMP) during cellular stress and infections. In these scenarios, it is known to exhibit several immunomodulatory functions, which also include antitumor immunity [45].

The diagnostic and prognostic role of PTMA has been well documented in a variety of human cancers like hepatocellular carcinoma [20,27], breast cancer [25], esophageal cancer [46], lung cancer [28], melanoma [47], colorectal cancer [30], urinary bladder cancer [48] etc. In one of our past study involving oral cancer, a strong association of PTMA with the aggressiveness of the malignancy was observed [26]. All these studies suggest PTMA as a key oncogenic player, prominently associated with worsening histological subtype and poor prognosis. Contrary to these, it was recently reported that PTMA can also act as a tumor suppressor in bladder cancer [49]. Therefore, this protein may act differently in various cancers. However, there is no study as per the best of our knowledge that demonstrates its involvement in glial tumors.

Herein, we investigated the role of PTMA in the progression and prognosis of glioma using institutional patient cohorts and online multiomics datasets. In agreement with previous reports, our immunostaining analysis also revealed discrete nuclear staining of PTMA in neoplastic astrocytes among various grades of gliomas [26,30]. Additionally, the nuclear PTMA staining intensity and percentage of immunostained cells increased significantly in a grade-wise manner to peak in GBM. In ovarian tissues, its expression has been shown to exhibit a positive correlation with cellular proliferation as measured by expression of Ki67 nuclear antigen [50] and proliferating cell nuclear antigen (PCNA) [51]. Intriguingly, we also observed a strong association between PTMA and Ki67 immunostaining. This trend of increased PTMA with the advancement of disease was observed both at the protein and mRNA levels. This finding is consistent with other cancers where PTMA expression increased progressively with histological advancement of the disease [27,46,52,53].

In the context of regulation of PTMA expression, we did not observe the association between mutation of EGFR, p53, and PTEN in GBM tissues. Interestingly we observed higher PTMA expression in IDH wildtype gliomas. Therefore, we examined whether promoter DNA methylation is associated with PTMA expression (Supplementary Figure S5). A significant negative correlation between DNA methylation levels of some CpG sites with PTMA expression was observed in both LGG and GBM. This suggests that PTMA expression is at least partly regulated by DNA methylation in glioma. Further studies are required to ascertain the role of epigenetic alterations in PTMA regulation. Moreover, recently it was demonstrated that a circular RNA, Hsa_circ_0004277 acts as an miR-512-5p sponge in colorectal cancer cells, leading to up-regulation of PTMA expression and, thereby cell proliferation [54].

Astrocytic gliomas, particularly GBM, represent the prototype of cancers that invariably exhibit poor prognosis and dismal OS. Timely follow-up coupled with prompt medical, surgical, and radiological interventions can prolong the life and delay the inevitable to some extent. Therefore, it becomes rational and critically important to explore molecules that could be linked with survival and predict the prognosis. In this respect, we demonstrated a strong association of PTMA with poor prognosis evidenced by alarmingly reduced survival in higher PTMA-expressing patients. This finding is in-line with malignancies like hepatocellular cancer [27], esophageal cancer [46], gall bladder carcinoma [29], colorectal cancer [30], and oral cancer [26], where elevated PTMA was strongly associated with unfavorable therapeutic outcomes. The encouraging results of survival analysis revealed an extremely high HRs make us contemplate PTMA as a potential glioma biomarker. Interestingly its immunostaining exhibited a remarkable positive correlation with Ki67 (MIB-1 index). Therefore, depending upon PTMA immunopositivity, patients could be conveniently classified into two distinct groups for monitoring treatment outcomes. Importantly, the association of PTMA with poor OS was independent of other clinical markers, including the immunohistochemistry-based assessment of IDH mutation, α-thalassemia/intellectual disability syndrome X-linked (ATRX). This indeed mandates its consideration as a worthful prognosticator in clinical settings. In our study, immunohistochemistry was performed to check PTMA protein expression, while for quantifying mRNA expression, we only picked 5 representative samples of each grade. Nevertheless, for validating the association of PTMA expression with poor disease outcomes, we used both, immunohistochemistry and gene expression datasets.

It is highly pertinent to identify underlying molecular features of glioma, which independently or in combination with established markers, can guide therapy and biomarker research. The recent emergence of high throughput sequencing and proteomics analysis has enhanced the understanding of glioma biology and also aided in the identification of novel biomarkers [55]. Numerous studies have utilized gene expression datasets from TCGA, CGGA etc., to identify potential prognostic biomarkers in glioma such as GINS4 [56], SOCS3 [57], PTPRN [58], PLAT, IGFBP2, BCAT1, SERPINH1 [59], MAGEH1 [55] etc. Some of these markers have also been validated further in the patient cohort and also by functional analysis [56,57]. The same datasets have been used in the present study to establish the utility of PTMA as an independent predictor of patient survival. Immunohistochemistry is widely preferred over gene expression analysis to determine molecular alterations and protein expression of biomarkers like IDH, p53, MIB-1, ATRX etc [60]. Therefore PTMA immunostaining holds promise for its integration into the clinical settings for the better overall management of gliomas. However, a further study in a larger cohort of glioma patients is warranted to validate the comparative prognostic utility of PTMA immunostaining and other such identified markers.

The utilization of highly characterized glioma datasets enabled us to determine a wide variety of clinically relevant associations of PTMA. However, further studies are required to determine the detailed molecular functions of this protein in glioma. In this regard, we performed pathway analysis using the list of genes with significant correlation to PTMA, which revealed enrichment of cell proliferation-associated pathways, in agreement with its observed association with the proliferative index. Additionally, some other enriched pathways, including p53 signaling and cell adhesion, are consistent with previous reports regarding molecular functions of PTMA in different malignancies [30,61,62]. In the context of immunity, extracellular PTMA acts as a pleiotropic biologic response modifier, as it regulates the activities of various immune cells, including neutrophils and T cells [45]. PTMA has been shown to regulate cell proliferation and induction of type I interferon immunomodulatory activity in human macrophages [63,64]. PTMA has also been reported to be expressed by a subclass of neutrophils N2, present in lung tissue with unknown functions [65].

Analysis of CGGA data revealed a strong association between PTMA mRNA expression and patient survival independent of tumor recurrence status. However, it could not be validated in our patients due to the unavailability of recurrent tumor specimens. It will also be worthwhile to determine the association between PTMA immunostaining with time to recurrence and response to therapy [66]. Furthermore, efforts in our lab are going on to determine the effect of genetic ablation of PTMA on oncological features in this malignancy, which will complement these results in a better way. Future studies exploring the clinical significance and molecular functions, including the immunomodulatory potential of PTMA, are therefore highly warranted.

## Supplementary Material

Supplementary Figures S1-S5

## Data Availability

All datasets used in the manuscript have been mentioned in the ‘Materials and methods’ section. Materials and raw data that support the findings of the present study can be made available from the corresponding author upon reasonable request.

## Abbreviations

ATRX: α-thalassemia/intellectual disability syndrome X-linked
CGGA: Chinese Glioma Genome Atlas
DFI: disease-free interval
DSS: disease-specific survival
GBM: glioblastoma
HR: hazard ratio
IDH: isocitrate dehydrogenase
IRS: immunoreactivity score
LGG: lower grade glioma
OS: overall survival
PFI: progression-free interval
PTMA: prothymosin-α
REMBRANDT: REpository for Molecular BRAin Neoplasia DaTa
TCGA: The Cancer Genome Atlas
TMZ: Temozolomide
WHO: World Health Organization
