# Supplementary material for: Overexpression of prothymosin-α in glioma is associated with tumor aggressiveness and poor prognosis
Source: Biosci Rep. 2022 Apr 19;42(4):BSR20212685. doi: 10.1042/BSR20212685 (PMC9069441; doi:10.1042/BSR20212685)
Supplement: Supplementary Figures S1-S5 [file BSR-2021-2685_supp.pdf]

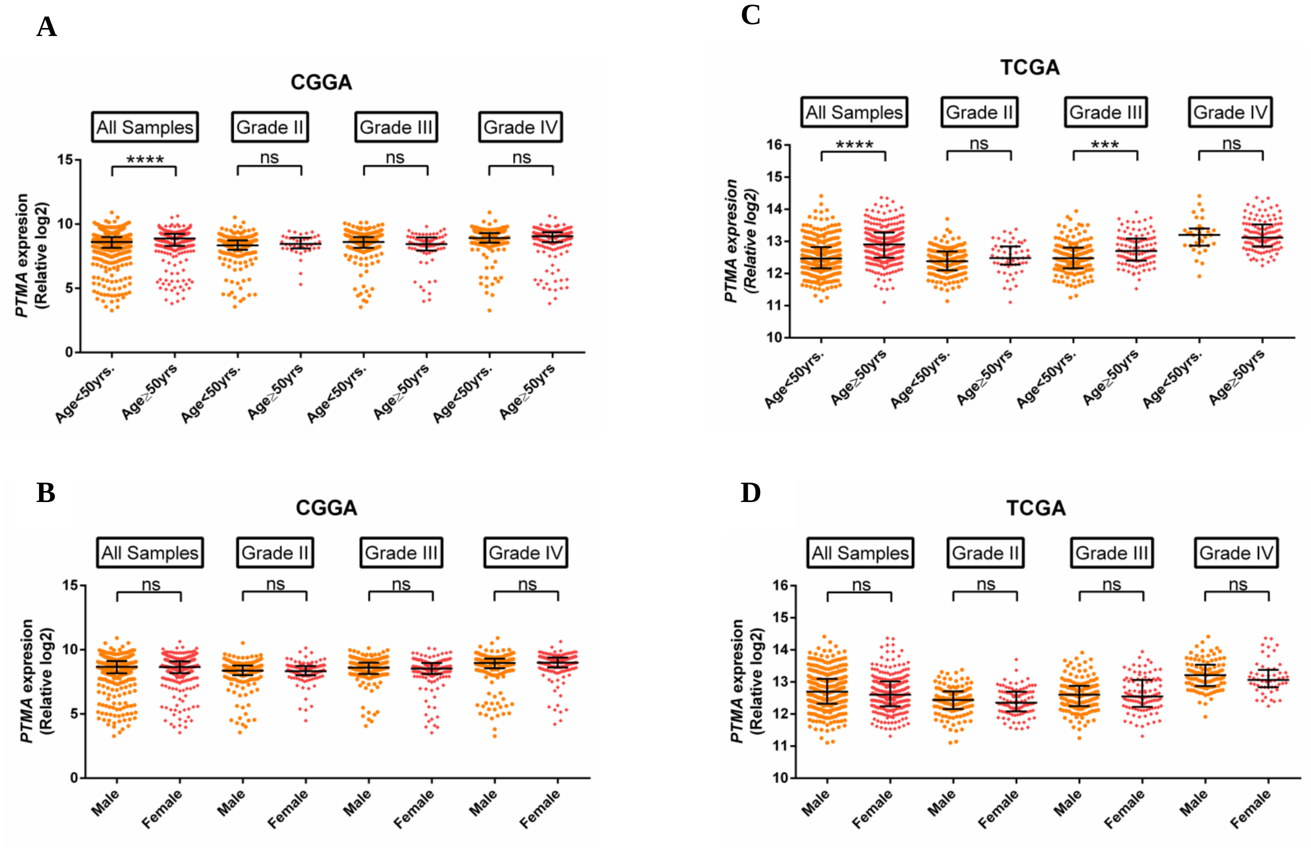

Supplementary Figure 1: Association of PTMA expression with age (A) and gender (B) in CGGA dataset and TCGA dataset (C and D) respectively.

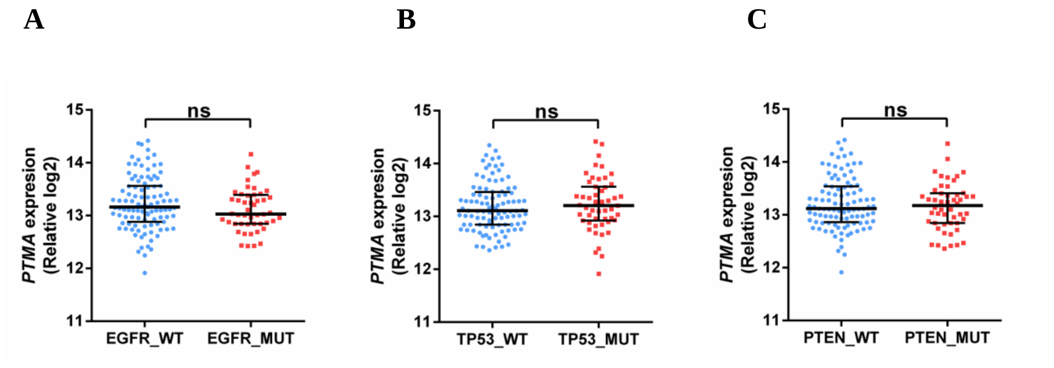

Supplementary Figure 2: Association of PTMA expression with molecular signatures in glioma datasets. (A) EGFR mutation status in TCGA-GBM (B) TP53 mutation status in TCGA-GBM (C) PTEN mutation status in TCGA-GBM.

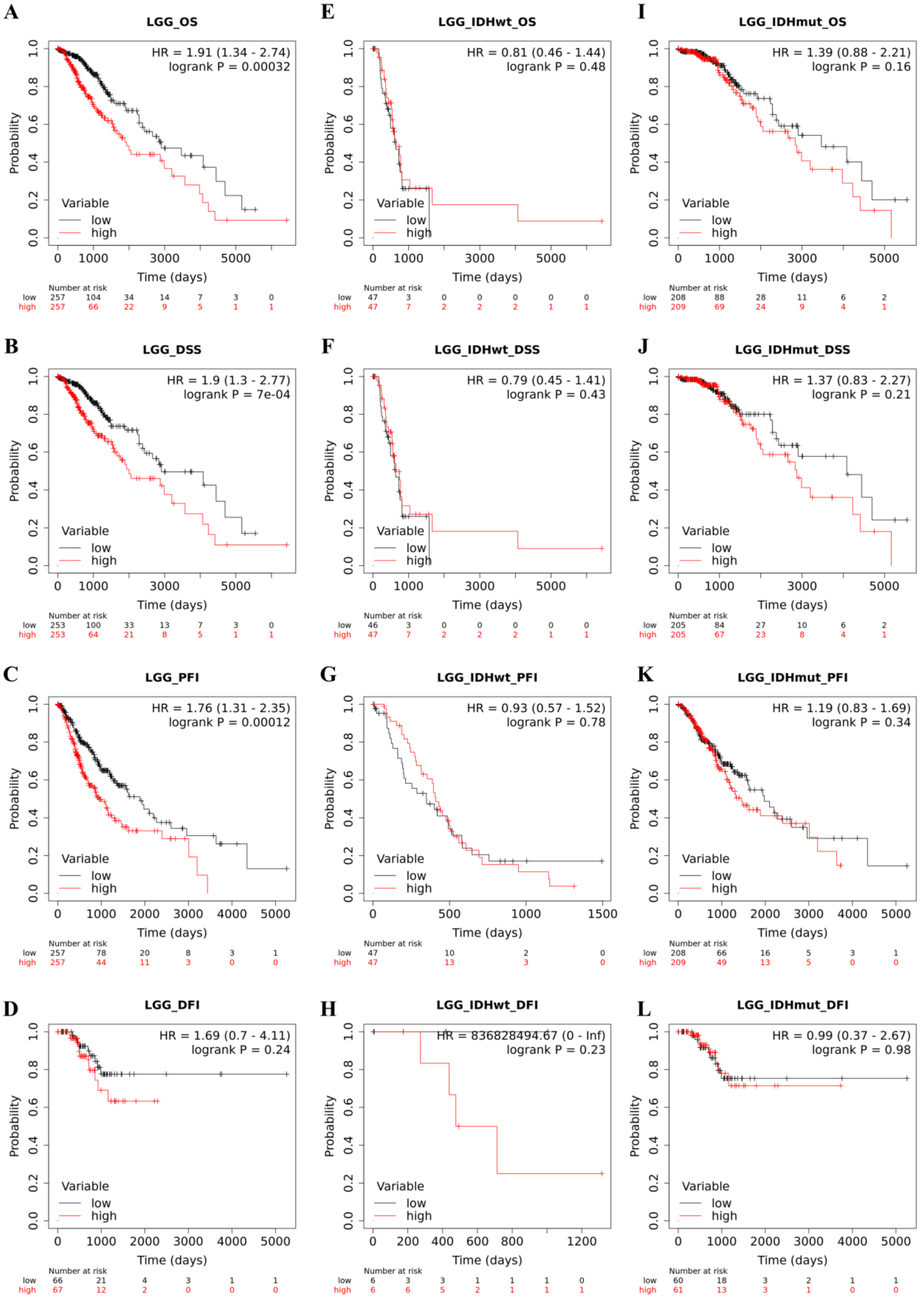

Supplementary Figure 3: Kaplan-Meier survival analysis showing overall survival (OS), disease-specific survival (DSS), progression-free interval (PFI), and disease-free interval (DFI), respectively in TCGA_LGG samples. (A-D) IDH mutant, (E-H) and IDH wild type (I-L). HR represents the hazard ratio.

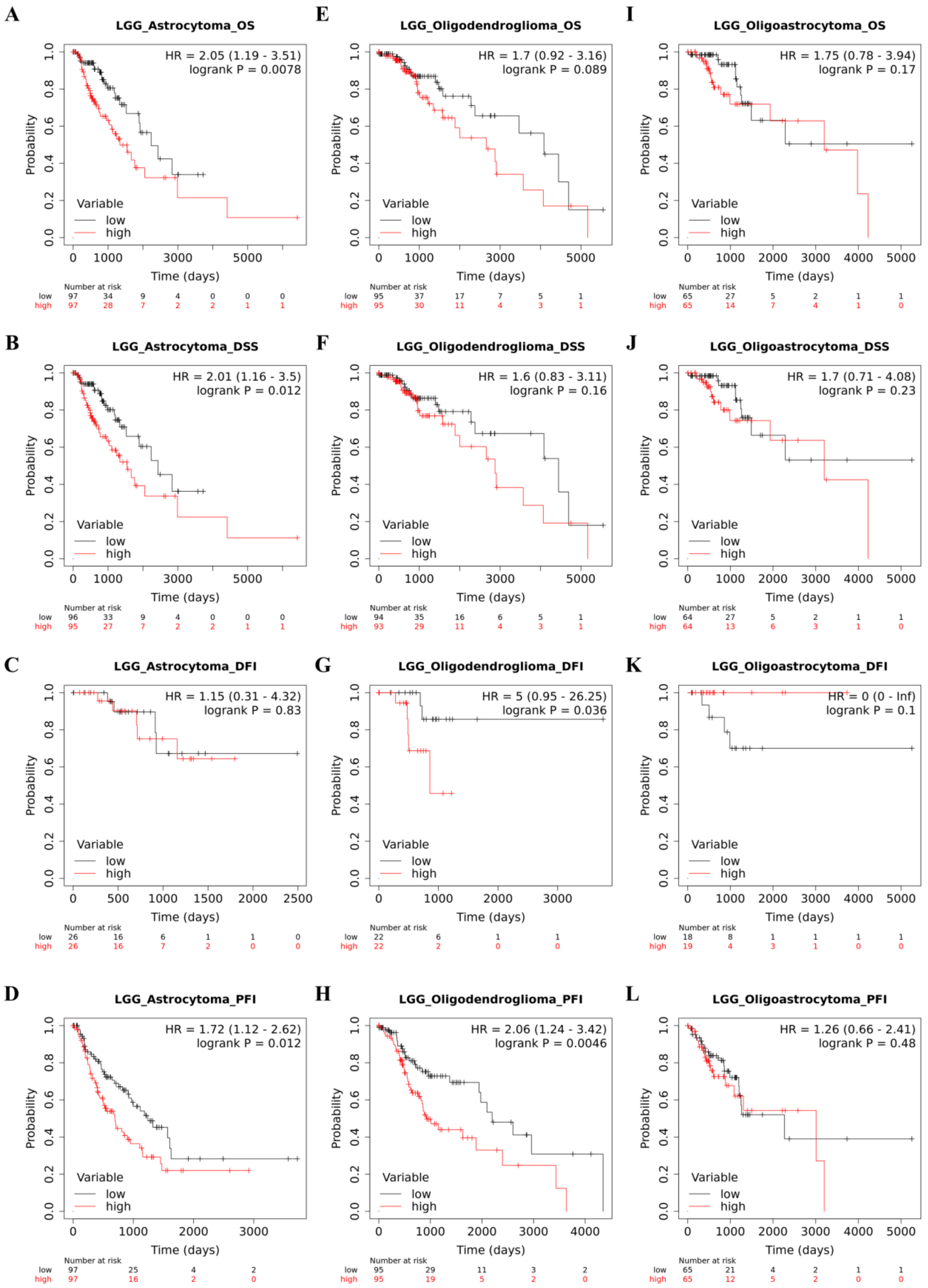

Supplementary Figure 4: Kaplan-Meier survival analysis showing OS, DSS, DFI, and PFI in TCGA_LGG based in tumor histology. (A-D) Astrocytoma; (E-H) Oligodendroglioma; (I-L) Oligoastrocytoma. HR represents the hazard ratio.

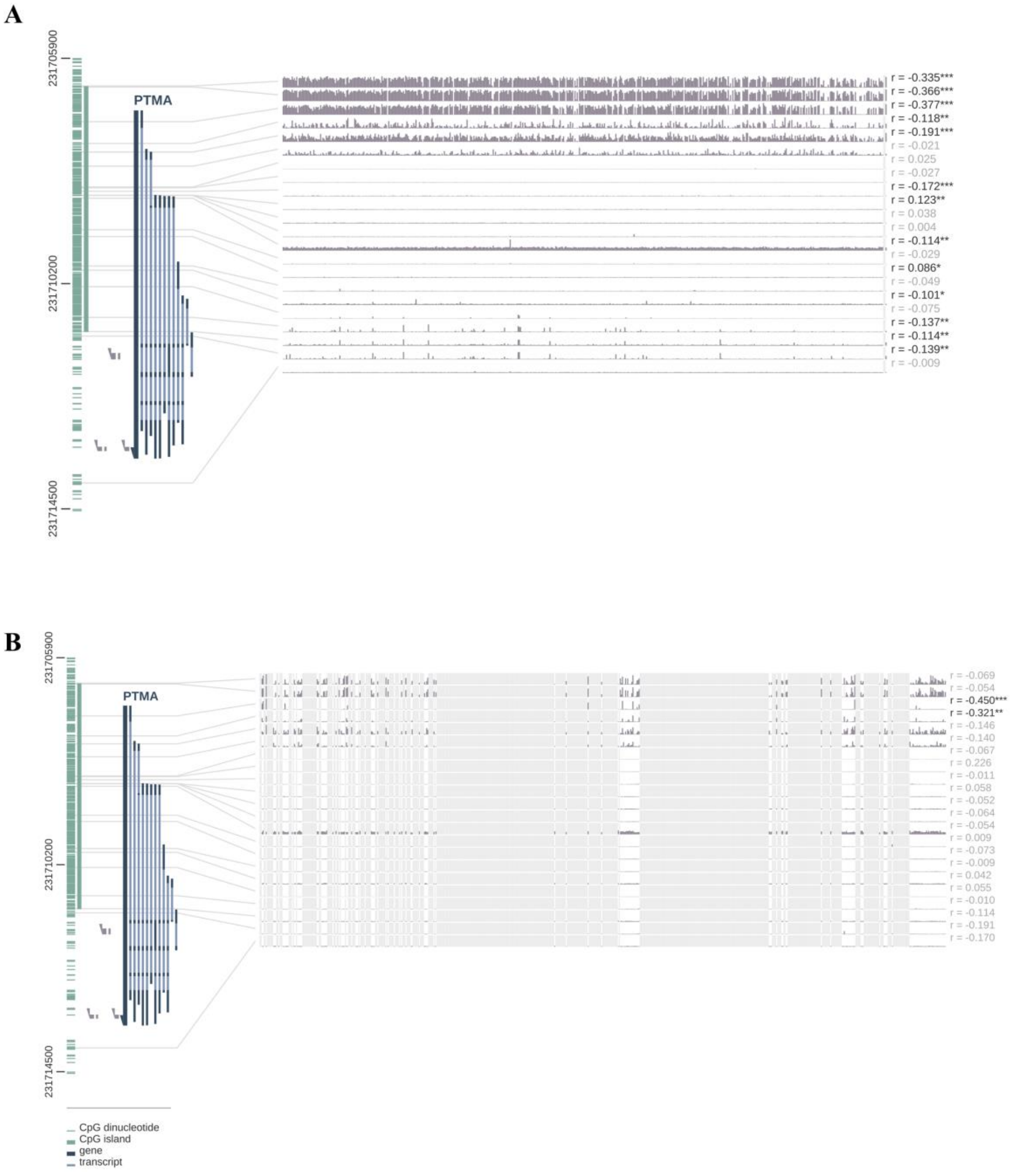

Supplementary Figure 5: Correlation of PAXX gene methylation with gene expression in (A) TCGA-LGG and (B) TCGA-GBM dataset from MEXPRESS webserver.

A)

B)

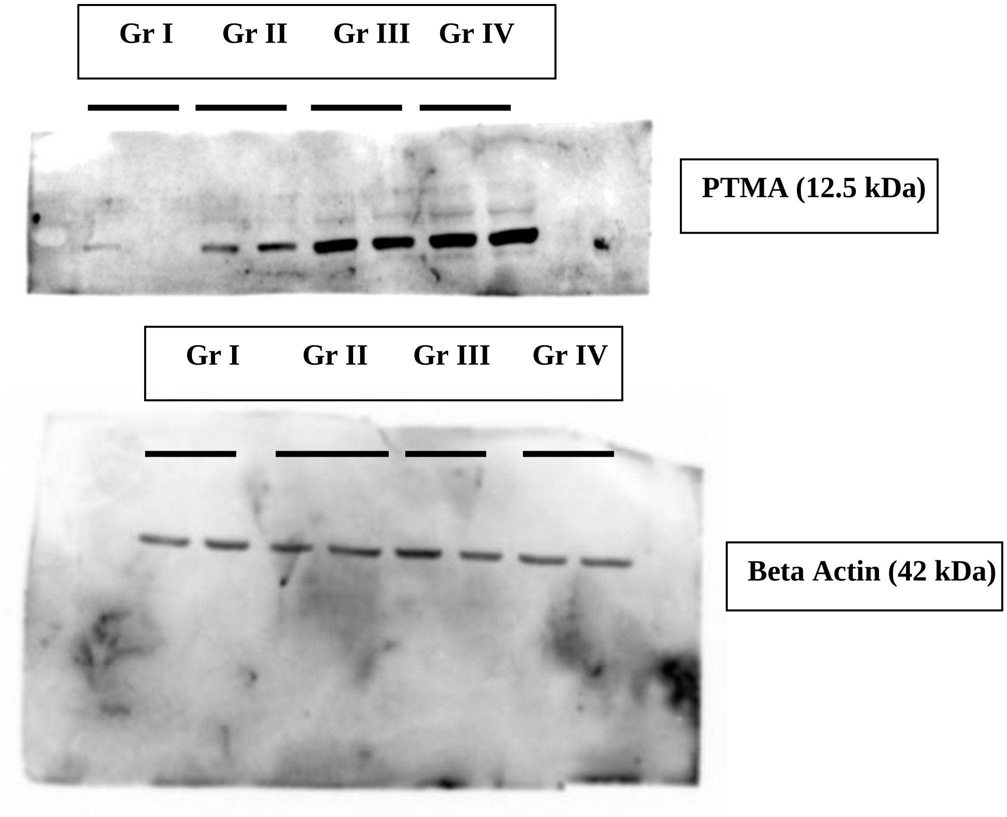

Supplementary Figure 6: Raw and uncropped western blot images of glioma tissue lysates (A and B), represented as figure 1E in main article file. Here, figure A and B represents blots for PTMA and beta actin respectively.
